# The monetary value of human lives lost due to neglected tropical diseases in Africa

**DOI:** 10.1186/s40249-017-0379-y

**Published:** 2017-12-18

**Authors:** Joses Muthuri Kirigia, Gitonga N. Mburugu

**Affiliations:** Meru University of Science and Technology, P.O. Box 972-60200, Meru, Kenya

**Keywords:** Neglected tropical diseases, Non-health gross domestic product loss, Value of life, Lost output approach, Human capital approach, Africa

## Abstract

**Background:**

Neglected tropical diseases (NTDs) are an important cause of death and disability in Africa. This study estimates the monetary value of human lives lost due to NTDs in the continent in 2015.

**Methods:**

The lost output or human capital approach was used to evaluate the years of life lost due to premature deaths from NTDs among 10 high/upper-middle-income (Group 1), 17 middle-income (Group 2) and 27 low-income (Group 3) countries in Africa. The future losses were discounted to their present values at a 3% discount rate. The model was re-analysed using 5% and 10% discount rates to assess the impact on the estimated total value of human lives lost.

**Results:**

The estimated value of 67 860 human lives lost in 2015 due to NTDs was Int$ 5 112 472 607. Out of that, 14.6% was borne by Group 1, 57.7% by Group 2 and 27.7% by Group 3 countries. The mean value of human life lost per NTD death was Int$ 231 278, Int$ 109 771 and Int$ 37 489 for Group 1, Group 2 and Group 3 countries, respectively. The estimated value of human lives lost in 2015 due to NTDs was equivalent to 0.1% of the cumulative gross domestic product of the 53 continental African countries.

**Conclusions:**

Even though NTDs are not a major cause of death, they impact negatively on the productivity of those affected throughout their life-course. Thus, the case for investing in NTDs control should also be influenced by the value of NTD morbidity, availability of effective donated medicines, human rights arguments, and need to achieve the NTD-related target 3.3 of the United Nations Sustainable Development Goal 3 (on health) by 2030.

**Electronic supplementary material:**

The online version of this article (10.1186/s40249-017-0379-y) contains supplementary material, which is available to authorized users.

## Multilingual abstracts

Please see Additional file 1 for translations of the abstract into the five official working languages of the United Nations.

## Background

Africa has 54 countries: 1 (1.9%) high-income, 9 (16.7%) upper-middle-income, 17 (31.5%) lower-middle-income and 27 (50%) low-income countries (see Table 1) [1]. The continent had a population of approximately 1 184 500 000 people in 2015 [2]. The total gross domestic product (GDP) for Africa that year was approximately International Dollars (Int$) 6 045 831 000 000 in 2015 [3].

**Table 1:** World Bank analytical classifications

Economic classification	Gross national income per capita in US$ (the World Bank’s fiscal year – 1 July 2015 to 30 June 2016)	Countries
High-income economies	> 12 475	Seychelles (1)
Upper-middle-income economies	4 036–12 475	Algeria, Angola, Botswana, Equatorial Guinea, Gabon, Libya, Mauritius, Namibia, South Africa (9)
Lower-middle-income economies	1 026–4 035	Cape Verde, Cameroon, Republic of Congo, Cote d’Ivoire, Djibouti, Egypt, Ghana, Kenya, Lesotho, Mauritania, Morocco, Nigeria, Sao Tome and Principe, Sudan, Swaziland, Tunisia, Zambia (17)
Low-income economies	≤ 1 025	Benin, Burkina Faso, Burundi, Central African Republic, Chad, Comoros, DRC, Eritrea, Ethiopia, The Gambia, Guinea, Guinea-Bissau, Liberia, Madagascar, Malawi, Mali, Mozambique, Niger, Rwanda, Senegal, Sierra Leone, Somalia, South Sudan, Tanzania, Togo, Uganda, Zimbabwe (27)

Source: World Bank [1]

Globally, an estimated total of 56 228 951 deaths from all causes occurred in 2015. Approximately 10 522 529 of those deaths happened in the African continent. Of these, 5 497 996 (52.2%) were from communicable, maternal, perinatal and nutritional conditions; 3 985 251 (37.9%) were from non-communicable diseases; and 1 039 282 (9.9%) were from injuries [4].

Out of the total global number of deaths, 206 155 resulted from NTDs, of which 67 860 (32.9%) occurred in Africa [4].

According to various reports [5–13], NTDs afflict mainly the poorest people in Africa, often with devastating effects for their entire life-course. They erode patients’ intellectual capacities, school attendance and educational performance, labour productivity and income earning potential, and thus aggravate and perpetuate inter-generational poverty among societies living in endemic areas [5–13].

In 2015, the United Nations (UN) General Assembly adopted resolution A/69/L.85 on the 2030 agenda for sustainable development. It contains 17 Sustainable Development Goals (SDGs), of which SDG 3 is centred on ensuring healthy lives and promoting wellbeing for all. The SDG has 13 targets, of which target 3.3 reads: *“By 2030, end the epidemics of AIDS, tuberculosis, malaria and neglected tropical diseases and combat hepatitis, water-borne diseases and other communicable diseases”* (p. 16) [14]. There is therefore an urgent need for collating economic burden evidence to use in advocacy in African countries among the ministries of finance, the private sector and development partners to increase investment towards global efforts to achieve the abovementioned target on ending the NTD epidemic.

Globally, a number of studies have been conducted on the economic burden of a single NTD [15–31] in one or a few countries [32, 33]. To date, no study has attempted to measure the value of human lives lost due to NTDs in all or the majority of countries in continental Africa. Therefore, the study reported in this paper was an attempt to contribute to bridging this knowledge gap.

The paper answers the question: What is the value of human lives lost due to NTDs in continental Africa? More specifically, the objective was to estimate the monetary value of human lives lost due to NTDs in Africa in 2015.

## Methods

### Study area and population

This study was conducted in the African continent, which has a total of 54 countries. Somalia was excluded from the study, as it did not have data on per-capita GDP and per-capita total health expenditure.

The study includes the following 16 NTDs as listed in the World Health Organization (WHO) Global Health Estimates 2015: African trypanosomiasis, schistosomiasis, leishmaniasis, lymphatic filariasis, cysticercosis, echinococcosis, dengue, rabies, ascariasis, leprosy, Chagas disease, trachoma, onchocerciasis, trichuriasis, hookworm disease and food-borne trematodes. There were no deaths reported in the WHO [4] source for the last six NTDs in African countries.

### Study design: The lost output approach or human capital approach (HCA)

The late Professor Gavin Mooney [34] outlined three types of approaches used in deriving monetary values for human life: (a) the implied values (or revealed preferences) approach, which is based on values implied by past healthcare decisions; (b) the HCA or lost output approach, which equates the value of human life with the value of livelihood; and (c) the willingness-to-pay (or contingent valuation) approach, which is based on how much individuals are prepared to pay to reduce the risk of morbidity or death. The strengths and weaknesses of each approach have been exhaustively discussed in Linnerooth [35], Mooney [36] and Jones-Lee [37].

The HCA or lost output approach was first applied by Petty [38]. However, its theoretical and practical underpinnings have been refined and enhanced by Fein [39], Mushkin and Collings [40], Weisbrod [41], and Landefeld and Seskin [42]. The approach has been widely applied in Asia-Pacific countries [43–53], North America [54–60] and Europe [61–66]. It has been applied in Africa to estimate the economic burdens of cholera [67], malaria [68, 69], HIV/AIDS [70, 71] and diabetes mellitus [72]. The specific approach used in the current study is similar to that developed and applied in estimating the indirect costs of child mortality [73], Ebola virus disease [74], tuberculosis [75] and maternal mortality [76] in the African region.

The choice of the HCA or lost output approach to place monetary values on years of human lives lost due to NTDs was based on successful past applications in estimating indirect costs of a number of health conditions in the region [73–76]; and availability of data on GDPs and total health expenditure per capita for all countries (except one) in Africa.

According to Mooney [34], this approach:

*“suggests that a life’s value can be measured in terms of the future expected life-time earnings of the individual concerned, adjusted to allow for working life expectancy, participation rates in the labour force, and various other factors. The value of life or, more accurately in this context, of livelihood is then obtained by discounting these future earnings to their present value as is usual in public and private investment decisions. For this reason, economists term it the ‘human capital’ approach.”* (p.7)

The GDP is a monetary measure of the market value of all final goods and services produced within a country in a specific period, e.g. yearly in our case [77, 78]. The NTD premature mortality impacts negatively on all the components of the GDP, including consumption expenditure, investment, government expenditure and net exports, i.e. exports less imports. We used per-capita GDP data to value the years of life lost (YLLs) to premature mortality from NTDs. The per-capita GDP is obtained by dividing the total GDP of a country by its population. The WHO [79] and Chisholm et al. [80] advise that when the focus of an economic burden of a disease study is on overall productivity losses, the quantity of interest should be the effect on the pooled output of remunerated and unremunerated labour as measured by non-health GDP.

The value of human lives lost (*VHLLost*) due to NTDs in Africa is equal to the sum of non-health GDP losses of the 53 countries. The *VHLLost*due to NTD deaths (NTDDs) in a country is the sum of the potential non-health GDP lost due to NTDDs among people aged 0–4 years (*VHLLost*_0 − 4_), 5–14 years (*VHLLost*_5 − 14_), 15–29 years (*VHLLost*_15 − 29_), 30–49 years (*VHLLost*_30 − 49_), 50–59 years *VHLLost*_50 − 59_, 60–69 years (*VHLLost*_60 − 69_), and 70 years and above *VHLLost*_≥70_ [73–76].

The *VHLLost*associated with NTDDs among persons of a specific age group equals the total discounted YLLs per-capita non-health GDP in purchasing power parity (PPP) and the total NTDDs for the age group [73, 74, 76]. Each country’s discounted value of human lives loss associated with NTDDs was appraised using the eqs. (1) to (8), as shown below:

1 $$ VHLLost=\left(\begin{array}{l}{VHLLost}_{0-4}+{VHLLost}_{5-14}+{VHLLost}_{15-29}+\\ {}{VHLLost}_{30-49}+{VHLLost}_{50-59}+{VHLLost}_{60-69}+{VHLLost}_{\ge 70}\end{array}\right) $$

2 $$ {\displaystyle \begin{array}{l}{VHLLost}_{0-4}=\sum \limits_{i=1}^k\left\{\left[1/{\left(1+r\right)}^k\right]\right.\times \left[{NHGDPPC}_{Int\$}\right]\times \left.\left[{NTDD}_{0-4}\right]\right\}=\\ {}\kern2.16em \left\{\left[1/{\left(1+r\right)}^1\right]\right.\times \left[{NHGDPPC}_{Int\$}\right]\times \left.\left[{NTDD}_{0-4}\right]\right\}+\\ {}\kern2.16em \left\{\left[1/{\left(1+r\right)}^2\right]\right.\times \left[{NHGDPPC}_{Int\$}\right]\times \left.\left[{NTDD}_{0-4}\right]\right\}+\dots +\\ {}\kern2.16em \left\{\left[1/{\left(1+r\right)}^k\right]\right.\times \left[{NHGDPPC}_{Int\$}\right]\times \left.\left[{NTDD}_{0-4}\right]\right\}\;\end{array}} $$

3 $$ {\displaystyle \begin{array}{l}{VHLLost}_{5-14}=\sum \limits_{i=1}^k\left\{\left[1/{\left(1+r\right)}^k\right]\right.\times \left[{NHGDPPC}_{Int\$}\right]\times \left.\left[{NTDD}_{5-14}\right]\right\}=\\ {}\kern2.16em \left\{\left[1/{\left(1+r\right)}^1\right]\right.\times \left[{NHGDPPC}_{Int\$}\right]\times \left.\left[{NTDD}_{5-14}\right]\right\}+\\ {}\kern2.16em \left\{\left[1/{\left(1+r\right)}^2\right]\right.\times \left[{NHGDPPC}_{Int\$}\right]\times \left.\left[{NTDD}_{5-14}\right]\right\}+\dots +\\ {}\kern2.16em \left\{\left[1/{\left(1+r\right)}^k\right]\right.\times \left[{NHGDPPC}_{Int\$}\right]\times \left.\left[{NTDD}_{5-14}\right]\right\}\end{array}} $$

4 $$ {\displaystyle \begin{array}{l}{VHLLost}_{15-29}=\sum \limits_{i=1}^k\left\{\left[1/{\left(1+r\right)}^k\right]\right.\times \left[{NHGDPPC}_{Int\$}\right]\times \left.\left[{NTDD}_{15-29}\right]\right\}=\\ {}\kern2.16em \left\{\left[1/{\left(1+r\right)}^1\right]\right.\times \left[{NHGDPPC}_{Int\$}\right]\times \left.\left[{NTDD}_{15-29}\right]\right\}+\\ {}\kern2.16em \left\{\left[1/{\left(1+r\right)}^2\right]\right.\times \left[{NHGDPPC}_{Int\$}\right]\times \left.\left[{NTDD}_{15-29}\right]\right\}+\dots +\\ {}\kern2.16em \left\{\left[1/{\left(1+r\right)}^k\right]\right.\times \left[{NHGDPPC}_{Int\$}\right]\times \left.\left[{NTDD}_{15-29}\right]\right\}\end{array}} $$

5 $$ {\displaystyle \begin{array}{l}{VHLLost}_{30-49}=\sum \limits_{i=1}^k\left\{\left[1/{\left(1+r\right)}^k\right]\right.\times \left[{NHGDPPC}_{Int\$}\right]\times \left.\left[{NTDD}_{30-49}\right]\right\}=\\ {}\kern2.16em \left\{\left[1/{\left(1+r\right)}^1\right]\right.\times \left[{NHGDPPC}_{Int\$}\right]\times \left.\left[{NTDD}_{30-49}\right]\right\}+\\ {}\kern2.16em \left\{\left[1/{\left(1+r\right)}^2\right]\right.\times \left[{NHGDPPC}_{Int\$}\right]\times \left.\left[{NTDD}_{30-49}\right]\right\}+\dots +\\ {}\kern2.16em \left\{\left[1/{\left(1+r\right)}^k\right]\right.\times \left[{NHGDPPC}_{Int\$}\right]\times \left.\left[{NTDD}_{30-49}\right]\right\}\end{array}} $$

6 $$ {\displaystyle \begin{array}{l}{VHLLost}_{50-59}=\sum \limits_{i=1}^k\left\{\left[1/{\left(1+r\right)}^k\right]\right.\times \left[{NHGDPPC}_{Int\$}\right]\times \left.\left[{NTDD}_{50-59}\right]\right\}=\\ {}\kern2.16em \left\{\left[1/{\left(1+r\right)}^1\right]\right.\times \left[{NHGDPPC}_{Int\$}\right]\times \left.\left[{NTDD}_{50-59}\right]\right\}+\\ {}\kern2.16em \left\{\left[1/{\left(1+r\right)}^2\right]\right.\times \left[{NHGDPPC}_{Int\$}\right]\times \left.\left[{NTDD}_{50-59}\right]\right\}+\dots +\\ {}\kern2.16em \left\{\left[1/{\left(1+r\right)}^k\right]\right.\times \left[{NHGDPPC}_{Int\$}\right]\times \left.\left[{NTDD}_{50-59}\right]\right\}\end{array}} $$

7 $$ {\displaystyle \begin{array}{l}{VHLLost}_{60-69}=\sum \limits_{i=1}^k\left\{\left[1/{\left(1+r\right)}^k\right]\right.\times \left[{NHGDPPC}_{Int\$}\right]\times \left.\left[{NTDD}_{60-69}\right]\right\}=\\ {}\kern2.16em \left\{\left[1/{\left(1+r\right)}^1\right]\right.\times \left[{NHGDPPC}_{Int\$}\right]\times \left.\left[{NTDD}_{60-69}\right]\right\}+\\ {}\kern2.16em \left\{\left[1/{\left(1+r\right)}^2\right]\right.\times \left[{NHGDPPC}_{Int\$}\right]\times \left.\left[{NTDD}_{60-69}\right]\right\}+\dots +\\ {}\kern2.16em \left\{\left[1/{\left(1+r\right)}^k\right]\right.\times \left[{NHGDPPC}_{Int\$}\right]\times \left.\left[{NTDD}_{60-69}\right]\right\}\end{array}} $$

8 $$ {\displaystyle \begin{array}{l}{VHLLost}_{\ge 70}=\sum \limits_{i=1}^k\left\{\left[1/{\left(1+r\right)}^k\right]\right.\times \left[{NHGDPPC}_{Int\$}\right]\times \left.\left[{NTDD}_{\ge 70}\right]\right\}=\\ {}\kern2.16em \left\{\left[1/{\left(1+r\right)}^1\right]\right.\times \left[{NHGDPPC}_{Int\$}\right]\times \left.\left[{NTDD}_{\ge 70}\right]\right\}+\\ {}\kern2.16em \left\{\left[1/{\left(1+r\right)}^2\right]\right.\times \left[{NHGDPPC}_{Int\$}\right]\times \left.\left[{NTDD}_{\ge 70}\right]\right\}+\dots +\\ {}\kern2.16em \left\{\left[1/{\left(1+r\right)}^k\right]\right.\times \left[{NHGDPPC}_{Int\$}\right]\times \left.\left[{NTDD}_{\ge 70}\right]\right\}\end{array}} $$

Where: 1/(1 + *r*)^*k*^ is the discount factor that converts future *VHLLost*into today’s dollars; *r* is an interest rate that measures the opportunity cost of lost livelihood or output; $$ \sum \limits_{i=1}^k $$ is the summation from year *i* to *k*; *i* is the first year of life lost, and *k* is the final year of the total number of YLLs per NTDD, which is obtained by subtracting the mean age at death for NTD-related causes from global maximum average life expectancy; *NHGDPPC*_*Int*$_ is per capita non-health GDP in PPP, which is obtained by subtracting per-capita total health expenditure (*PCTHE*) from per-capita GDP (*GDPPC*_*Int*$_ ); *NTDD*_0 − 4_ is the number of NTDDs among those aged 0–4 years in country *m* in 2015; *NTDD*_5 − 14_ is the number of NTDDs among those aged 5–14 years in country *m* in 2015; *NTDD*_15 − 29_ is the number of NTDDs among those aged 15–29 years in country *m* in 2015; *NTDD*_30 − 49_ is the number of NTDDs among those aged 30–59 years in country *m* in 2015; *NTDD*_50 − 59_is the number of NTDDs among those aged 50–59 years in country *m* in 2015;*NTDD*_60 − 69_is the number of NTDDs among those aged 60–69 years in country *m* in 2015; and *NTDD*_≥70_ is the number of NTDDs among those aged 70 years and above in country *m* in 2015. The base year to which future losses in value of human life were discounted was 2015. The discount factor used for losses occurring at diverse years hinge on both the number of years, *k*_,_ over which discounting is done and the discount rate (r) [76, 81–83].

### Data sources

The abovementioned eight equations were estimated using data from the WHO, International Labour Organization (ILO) and International Monetary Fund (IMF) sources. The parameters used in the analysis and data sources are summarised in Table 2.

### Data analysis

The analysis was done using Excel software (Microsoft, New York) following a number of steps:

- *Step 1*: The countries were categorised into three economic groups for comparative purposes, as shown in Table 2. Group 1: 10 high- and upper-middle-income countries; Group 2: 17 lower-middle-income countries; and Group 3: 27 low-income countries [1].
- *Step 2:* The eight formulas outlined above were built into a spreadsheet for each of the 53 countries.
- *Step 3:* The NTDDs by country and age brackets were downloaded from the WHO Global Health Estimates 2015 [4] (see Additional file 2). The methodological details of how the number of deaths for each NTD by age bracket per country was calculated are detailed in a WHO source [84].
- *Step 4:* The life expectancy data used for all countries were sourced from Table 2.1 of the WHO document entitled “WHO methods and data sources for global burden of disease estimates 2000 – 2015” [85] (see Additional file 3). Thus, following this estimation, instead of using individual African country’s life expectancies, we used the highest global projected life expectancies achieved by women in Japan and the Republic of Korea, with a life expectancy at birth of 91.9 years [85]. According to the WHO, this represents the maximum life span of an individual in good health who is not exposed to avoidable health risks or severe injuries, and receives appropriate health services. The WHO [85] provides maximum life spans for 20 age groups, while our study has seven age groups. The maximum life spans (in years) for the seven age groups under consideration in this study were obtained as follows:

- 0–4 is the average of the highest global life spans for neonatal (91.93 years), post-neonatal (91.55 years) and 1–4 years (89.41 years), i.e. (91.93 + 91.55 + 89.41)/3 = 90.96 years;
- 5–14 is the average of the highest global life spans for those aged 5–9 and 10–14 years, i.e. (84.52 + 79.53)/2 = 82.025 years;
- 15–29 is the average of the highest global life spans for those aged 15–19, 20–24 and 25–29 years, i.e. (74.54 + 69.57 + 64.6)/3 = 69.57 years;
- 30–49 is the average of the highest global life spans for those aged 30–34, 35–39, 40–44 and 45–49 years, i.e. (59.63 + 54.67 + 49.73 + 44.81)/4 = 52.21 years;
- 50–59 is the average of the highest global life spans for those aged 50–54 and 55–59 years, i.e. (39.92 + 35.07)/2 = 37.495 years;
- 60–69 is the average of the highest global life spans for those aged 60–64 and 65–69 years, i.e. (30.25 + 25.49)/2 = 27.87 years;
- 70+ years is the average of the highest global life spans for those aged 70–74, 75–79, 80–84 and 80 + years, i.e. (20.77 + 16.43 + 12.51 + 7.6)/4 = 14.3275 years.

**Table 2:** Parameters used in the analysis

Variable	Indicator	Sources
Mortality in 2015	Numbers and ratios of NTDDs occurring in the seven age brackets, per country	WHO [4]
YLLs	Global maximum YLLs for each of the seven age brackets	WHO [85]
Legal minimum age for employment	15 years	ILO [86]
Population in 2015	Population per country	WHO [2]
Average economic output per person in each of the 53 African countries with data in 2015	GDP per capita per country	IMF [3]
Expenditure on health in 2015	Projected 2015 total expenditure on health (THE) per person per country in Africa (projected using 2013 and 2014 THE data)	WHO [87]

Given that the legal minimum working age is 15 years, as according to the ILO [86], only the years above 14 were considered when calculating the productive YLLs for the 0–4 and 5–14 years’ age brackets.

- *Step 5:* The YLLs obtained in Step 4 were discounted at a discount rate of 3%.
- *Step 6:* The national and per-capita GDP in Int$ (or PPP) were downloaded from the IMF website [3].
- *Step 7:* The non-health per-capita GDP in Int$ or PPP (NHGDPPC) was estimated (see Additional file 4). The NHGDPPC was obtained by subtracting per-capita total health expenditure from per-capita GDP [87].
- *Step 8:* Sensitivity analysis was conducted. This study used a 3% discount rate, which has been used in past economic evaluation and health systems studies [67, 73, 84, 85, 88–90]. In order to gauge the sensitivity of the value of human life estimates to discount rate, eqs. (1) to (8) were re-estimated with 5% and 10% discount rates. Those equations were subsequently re-estimated assuming Africa’s maximum life expectancy of 75.6 years (i.e. life expectancy for Algeria) for all countries instead of their actual life expectancies to determine the impact on the value of human life estimates.
- *Step 9:* Each country’s population and NTDDs were sorted into three economic groups, i.e. Group 1, Group 2 and Group 3 (see Table 3).
- *Step 10:* The value of human life estimates for various countries were grouped into the three groups and descriptive statistics were calculated.
- The process of estimating value of human life lost due to NTDDs is illustrated in Additional file 5 using actual data on Egypt.

**Table 3:** Total population and NTDDs by economic group in Africa

Economic class	Population in 2015	NTDDs in 2015
High-income and upper-middle-income countries (Group 1)	134 117 000	3 225
Lower-middle-income countries (Group 2)	509 004 000	26 888
Low-income countries (Group 3)	541 379 000	37 747
TOTAL	1 184 500 000	67 860

Source: WHO [2]

### Ethics approval and consent to participate

This study did not require approval from the Meru University of Science and Technology Institutional Research Ethics Review Committee because it did not involve the use of any animal, or human data or tissue. In addition, the study did not involve any participation of human beings. It was based completely on secondary statistical data published on the WHO, World Bank and IMF websites.

## Results

An estimated 67 860 (1.23%) of communicable, maternal, perinatal and nutritional conditions deaths resulted from 16 NTDs [4]. About 8.1% of these deaths occurred among those aged 0–4 years, 16.7% among those aged 5–14 years, 21.4% among those aged 15–29 years, 23.8% among those aged 30–49 years, 10.1% among those aged 50–59 years, 10.2% among those aged 60–69 years, and 9.7% among those aged 70 years and above. Thus, 55.3% of NTDDs occurred among the most productive age bracket of 15–59 years.

About 4.75% of the NTDDs were borne by the high- and upper-middle-income countries (Group 1), 39.62% by the lower-middle-income countries (Group 2) and 55.62% by the low-income countries (Group 3). The mean NTDDs per country was 1257 (STD = 2329); varying from a minimum of 1 in Seychelles to a maximum of 13 944 in Nigeria. The non-health GDP per capita in the continent was Int$ 5724 (STD = 7233), ranging from Int$ 739 in the Democratic Republic of the Congo (DRC) to Int$ 37 598 in Equatorial Guinea. The continental mean total expenditure on health in 2015 was Int$ 315 (STD = 325), varying from Int$ 24.7 in Madagascar to Int$ 1200 in South Africa.

### Value of human life loss attributable to NTDs

The 67 860 NTDDs led to a loss of human life worth Int$ 5 112 472 607; which is approximately 0.1% of the Continent’s GDP in 2015 (see Table 4). Almost 14.6% of the loss was suffered by Group 1, 57.7% by Group 2 and 27.7% by Group 3 countries. The mean value of human life lost was Int$ 75 339 per NTDD. The expected value of human lives lost across the continent varied widely, from Int$ 185 766 in Sao Tome and Principe to Int$ 1 625 450 009 in Nigeria. The potential value of human lives lost was under Int$ 10 million in 16 countries, between Int$ 10 million and Int$ 50 million in 21 countries, between Int$ 51 million and Int$ 100 million in one country, and over Int$ 100 million in 15 countries.

**Table 4:** Present value of human lives lost due to NTDDs in Africa (Int$ or PPP, in 2015)

Summary of indirect costs	High-income and upper-middle- income countries Sub-total cost (Int$)	Lower-middle- income countries Sub-total cost (Int$)	Low-income countries Sub-total cost (Int$)	Grand total cost (Int$)
(1). Total present value of NTDDs	745 815 366	2 951 569 697	1 415 087 545	5 112 472 607
(2). Average present value per NTDD	231 278	109 771	37 489	75 339
(3). Average present value per person in population	5.6	5.8	2.6	4.3
% of grand total	14.6	57.7	27.7	100

Out of the total loss in the entire continent, 19.1% was borne by those aged 0–4 years, 21.9% by those aged 5–14 years, 28.4% by those aged 15–29 years, 25.8% by those aged 30–49 years, 4% by those aged 50–59 years, 0.5% by those aged 60–69 years, and 0.2% by those aged 70 years and above. Thus, those in the most productive age bracket of 15–59 years bore 58.2% of the losses.

### Value of human life lost among group 1 countries

**Fig. 1:**
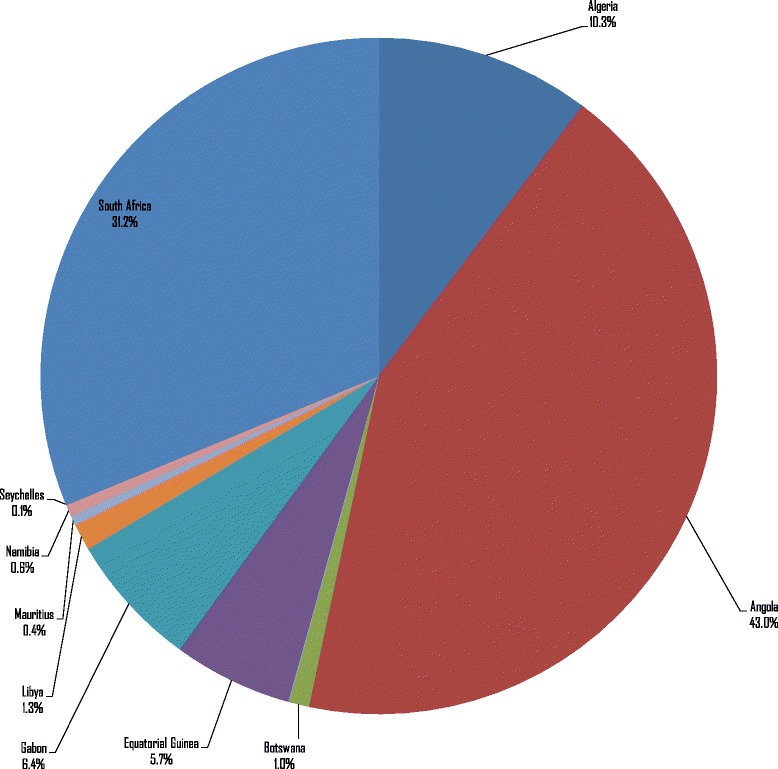
Value of human lives lost due to NTDDs in high-income and upper-middle-income countries (Group 1) of Africa (Int$, in 2015)

The 3225 NTDDs in Group 1 countries resulted in an expected loss of Int$ 745 815 366 in terms of the value of human life in 2015, which was equal to 0.04% of the group’s total GDP. The total value of human lives lost varied greatly, from Int$ 496 645 in Seychelles to Int$ 321 053 534 in Angola. Figure 1 shows the distribution of Group 1’s total value of human lives lost. Approximately 74.2% of the loss was borne by Angola and South Africa.

Group 1’s total present value of human life lost was distributed as follows across the NTDs: 42% schistosomiasis, 25.8% cysticercosis, 15.6% rabies, 5.2% ascariasis, 3.7% African trypanosomiasis, 3.3% leprosy, 2.3% leishmaniasis, 1.2% dengue, and 0.9% echinococcosis (see Table 5). Thus, the first three diseases accounted for 83.3% of losses incurred by Group 1.

**Table 5:** Distribution of present value of human lives lost in Africa across economic groups and NTDs (in 2015 Int$ or PPP)

NTDs	Group 1 Deaths in 2015	Group 1 Present Values in 2015 INT$	%	Group 2 Deaths in 2015	Group 2 Present Values in 2015 INT$	%	Group 3 Deaths in 2015	Group 3 Present Values in 2015 INT$	%
African Trypanosomiasis	122	28 205 045	3.8	157	17 233 681	0.6	5132	192 392 224	13.6
Chagas disease	–	–	–	–	–	–	–	–	–
Schistosomiasis	1354	313 029 760	42.0	10 798	1 185 282 070	40.2	10 645	399 067 659	28.2
Leishmaniasis	74	17 107 978	2.3	2467	270 799 302	9.2	3331	124 875 000	8.8
Lymphatic filariasis	–	–	–	–	–	–	–	–	–
Onchocerciasis	–	–	–	–	–	–	–	–	–
Cysticercosis	832	192 349 158	25.8	6610	725 570 891	24.6	8907	333 912 225	23.6
Echinococcosis	28	6 473 289	0.9	176	19 319 285	0.7	189	7 085 372	0.5
Dengue	39	9 016 367	1.2	353	38 748 340	1.3	311	11 658 999	0.8
Trachoma	–	–	–	–	–	–	–	–	–
Rabies	504	116 519 202	15.6	4835	530 731 507	18.0	6883	258 035 011	18.2
Ascariasis	167	38 608 545	5.2	932	102 304 398	3.5	604	22 643 200	1.6
Trichuriasis	–	–	–	–	–	–	–	–	–
Hookworm disease	–	–	–	–	–	–	–	–	–
Food-borne trematodes	–	–	–	–	–	–	–	–	–
Leprosy	106	24 506 023	3.3	561	61 580 222	2.1	1745	65 417 855	4.6
TOTAL	3225	745 815 366	100	26 888	2 951 569 697	100	37 747	1 415 087 545	100

### Value of human life lost among group 2 countries

**Fig. 2:**
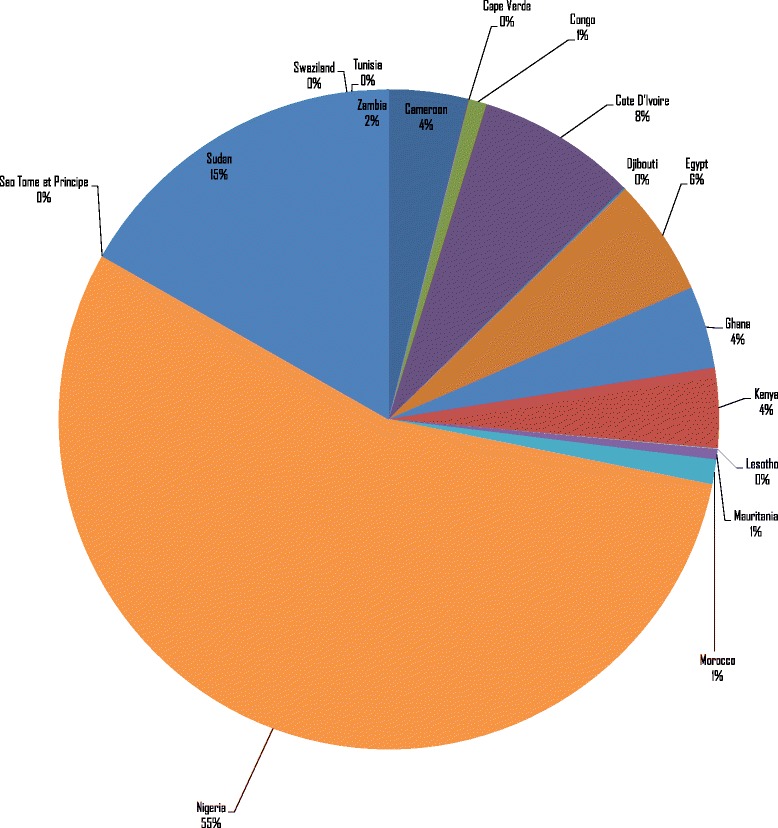
Value of human lives lost due to NTDDs in lower-middle-income countries (Group 2) of Africa (Int$, in 2015)

The 26 888 NTDDs in Group 2 countries resulted in an expected total loss of Int$ 2 951 569 697 in the value of human life in 2015, or 0.09% of the group’s total GDP. The loss varied from Int$ 185 766 in Sao Tome and Principe to Int$ 1 625 450 009 in Nigeria. Figure 2 shows the distribution of Group 2’s total value of human lives lost. Approximately 55.1% of Group 2’s expected loss was borne by Nigeria alone. About 83% of Group 2’s expected loss was borne by Cote d’Ivoire, Egypt, Nigeria and Sudan.

Group 2’s total present value of human life lost was distributed as follows across the NTDs: 40.0% schistosomiasis, 24.6% cysticercosis, 18% rabies, 9.2% leishmaniasis, 3.5% ascariasis, 2.1% leprosy, 1.3% dengue, 0.7% echinococcosis, and 0.6% African trypanosomiasis. Therefore, the first three diseases were responsible for 82.6% of the Group 2 losses (see Table 5).

### Value of human life lost among group 3 countries

**Fig. 3:**
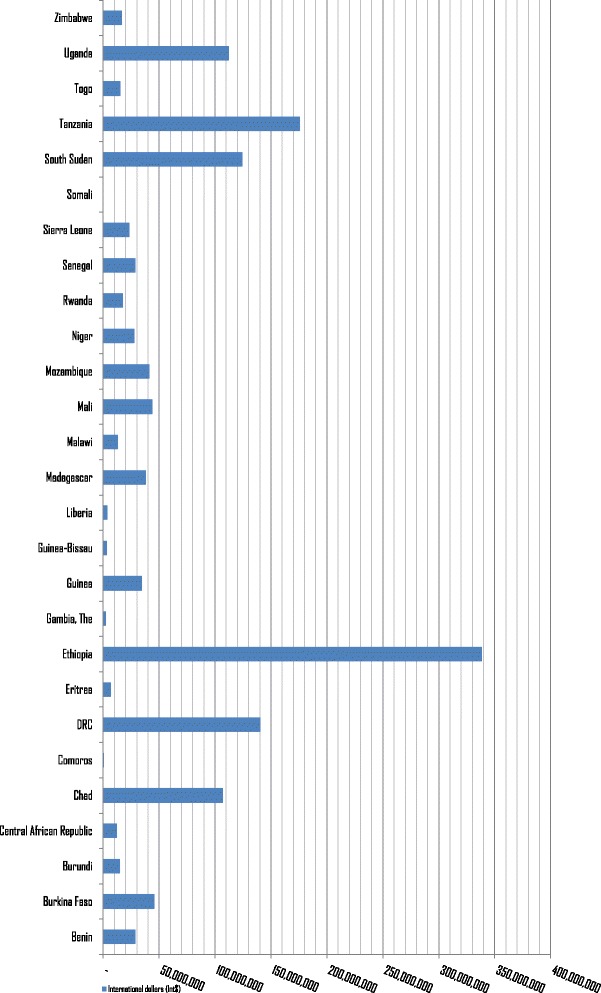
Value of human lives lost due to NTDDs in low-income countries (Group 3) of Africa (Int$, in 2015)

The estimated 37 747 NTDDs that occurred among Group 3 countries led to a total expected loss in value of human life of Int$ 1 415 087 545 in 2015, which is equivalent to 0.2% of the group’s total GDP. The expected loss ranged from Int$ 725 391 in Comoros to Int$ 338 485 350 in Ethiopia, which bore 23.9% of the group’s loss. The distribution of Group 3’s total value of human lives lost is depicted in Fig. 3. The DRC, Ethiopia, South Sudan, Tanzania and Uganda together accounted for 63% of the expected loss in this group. It is interesting to note that although Group 3 had 10 000 more NTDDs than Group 2, the value of human lives lost of Group 2 was higher than that of Group 3 by Int$ 1.54 billion because Group 2 had higher per-capita GDP.

Group 3’s total present value of human life lost was distributed as follows across the NTDs: 28.2% schistosomiasis, 23.7% cysticercosis, 18.2% rabies, 13.6% African trypanosomiasis, 8.8% leishmaniasis, 4.6% leprosy, 1.6% ascariasis, 0.8% dengue and 0.5% echinococcosis. Thus, the first three diseases were responsible for 70% of Group 3’s losses (see Table 5).

### Average value of human life losses

The mean value of human lives lost per NTDD and per person in the population for the 53 countries are displayed in Table 6. These values were obtained by dividing each group’s total value of human life lost by its total NTDDs. The mean value of human life lost per person in the population for each group was calculated by dividing the group’s total value of human life lost by its population.

**Table 6:** Discounted value of human lives lost due to NTDDs among continental Africa countries (Int$ or PPP, in 2015)

Country	(A) Population	(B) NTDDs	(C) Total value of all human lives lost due to NTDs (Int$)	(D) Value of human life lost per NTDD (Int$) [D = (C/B)]	(E) Value of human life lost per person in population (Int$) [E = (C/A)]
Algeria	39 667 000	25	76 687 914	304 735	1.9
Angola	25 022 000	1854	321 053 534	173 188	12.83
Benin	10 880 000	599	28 473 736	47 543	2.62
Botswana	2 262 000	22	7 430 499	341 854	3.28
Burkina Faso	18 106 000	1088	45 761 966	42 074	2.53
Burundi	11 179 000	751	14 673 682	19 547	1.31
Cameroon	23 344 000	1505	115 191 263	76 543	4.93
Cape Verde	521 000	4	642 650	149 761	1.23
Central African Republic	4 900 000	742	12 132 085	16 357	2.48
Chad	14 037 000	1659	106 702 194	64 305	7.60
Comoros	788 000	20	727 391	35 531	0.92
Congo Republic of	4 620 000	175	26 065 572	148 966	5.64
Cote d’Ivoire	22 702 000	2758	231 097 654	83 803	10.18
DRC	77 267 000	7298	140 486 238	19 250	1.82
Equatorial Guinea	845 000	47	42 493 865	895 595	50.29
Eritrea	5 228 000	279	6 786 209	24 350	1.30
Ethiopia	99 391 000	7315	338 483 350	46 275	3.41
Gabon	1 725 000	107	47 986 750	447 687	27.82
Gambia The	1 991 000	68	2 574 353	37 974	1.29
Ghana	27 410 000	1150	119 008 952	103 489	4.34
Guinea	12 609 000	1271	34 402 618	27 069	2.73
Guinea-Bissau	1 844 000	95	3 193 144	33 478	1.73
Kenya	46 050 000	1469	114 657 578	78 047	2.49
Lesotho	2 135 000	29	1 897 047	66 004	0.89
Liberia	4 503 000	192	3 459 176	18 030	0.77
Madagascar	24 235 000	1017	38 131 704	37 481	1.57
Malawi	17 215 000	554	13 198 778	23 832	0.77
Mali	17 600 000	1009	43 813 514	43 425	2.49
Mauritania	4 068 000	154	14 928 594	97 022	3.67
Mauritius	1 273 000	7	3 212 952	441 942	2.52
Mozambiqu	27 978 000	1543	41 035 562	26 597	1.47
Namibia	2 459 000	18	4 305 273	234 472	1.75
Niger	19 899 000	1428	27 871 413	19 517	1.40
Nigeria	182 202 000	13 944	1 625 450 009	116 572	8.92
Rwanda	11 610 000	408	17 313 494	42 472	1.49
Sao Tome and Principe	190 000	3	185 766	65 785	0.98
Senegal	15 129 000	474	28 758 809	60 629	1.90
Seychelles	96 000	1	496 645	683 843	5.17
Sierra Leone	6 453 000	665	23 115 300	34 786	3.58
South Africa	54 490 000	882	232 433 738	263 625	4.27
South Sudan	12 340 000	3013	124 210 140	41 228	10.07
Swaziland	1 287 000	23	4 630 328	197 374	3.60
Tanzania	53 470 000	2278	175 770 061	77 176	3.29
Togo	7 305 000	443	15 077 774	34 028	2.06
Uganda	39 032 000	2344	112 108 332	47 836	2.87
Zambia	16 212 000	618	48 509 819	78 483	2.99
Zimbabwe	15 603 000	346	16 826 522	48 696	1.08
Djibouti	888 000	34	2 285 624	67 853	2.57
Egypt	91 508 000	630	169 948 068	269 620	1.86
Libya	6 278 000	35	9 714 196	278 383	1.55
Morocco	34 378 000	175	35 758 333	204 524	1.04
Sudan	40 235 000	4184	432 348 755	103 325	10.75
Tunisia	11 254 000	34	8 963 687	266 963	0.80

The value of human life lost per NTDD was Int$ 231 278 for Group 1, Int$ 109 771 for Group 2 and Int$ 37 489 for Group 3. The mean value of human life lost per person in the population was Int$ 5.6 for Group 1, Int$ 5.8 for Group 2 and Int$ 2.6 for Group 3 (see Table 4). The mean value of human life lost per NTDD in Group 1 was over two times that of Group 2 and almost six times that of Group 3.

The main determinant of expected value of human life lost is the magnitude of GDP per person. For instance, even if deaths in Group 1 countries such as Botswana, Libya, Mauritius and Seychelles were only 22, 35, 7 and 1, respectively, the value of human life lost per NTDD for these countries were considerable at Int$ 341 854 for Botswana, Int$ 278 383 for Libya, Int$ 441 942 for Mauritius and Int$ 683 843 for Seychelles. Group 3 countries with comparatively higher number of NTDDs such as for the DRC with 7298 deaths, Ethiopia with 7315 deaths, South Sudan with 3013 deaths and Uganda with 2344 deaths have relatively lower values of human life lost per NTDD of Int$ 19 250, Int$ 46 275, Int$ 41 228 and Int$ 47 836, respectively.

### Sensitivity analysis results

The use of a 5% discount rate resulted in a reduction in the total value of human life loss of Int$ 1 446 559 015 (28.3%) and the mean value of human life loss per NTDD by Int$ 21 317. Application of a 10% discount rate reduced the overall total value of human life loss by Int$ 3 030 347 733 (59.3%) and the mean value of human life loss per NTDD by Int$ 44 656.

## Discussion

The estimated total value of human life loss attributed to NTDDs is about 0.1% of the 2015 GDP of the 53 African countries. As demonstrated by the sensitivity analysis, the magnitude of the total value of human life loss hinges on the discount rate [73–75].

The total value of human life loss attributed to NTDDs is higher than the Int$ 1.69 billion for diabetes in Africa [72]. However, it was lower than that the Int$ 5.53 billion for maternal mortality and Int$ 50.4 billion for tuberculosis deaths in Africa [75, 76].

It may be argued that there is not much point to continue investing in NTDs, which cause a relatively lower number of deaths and productivity losses compared to, for example, maternal mortality and tuberculosis mortality. Such critiques should take into account that while NTDs are not a major cause of death, they have life-long debilitating effects on health-related quality of life of populations living in endemic areas [91–95], educational achievements of children, worker productivity and agricultural outputs [6, 92, 96, 97].

There are six main arguments for continued (and probably increased) investment to control, eliminate and eventually eradicate NTDs. First, in line with the 1948 UN Universal Declaration of Human Rights, it is the right of every person living in NTD-endemic areas to have unconstrained access to all effective preventive and treatment interventions [98, 99]. Thus, we concur with Molyneux [100] that the *“continuing advocacy for the relevance of control or elimination of NTDs must be placed in the context of universal health coverage and access to donated essential medicines for the poor as a [human] right”* (p. 1).

Second, the resources required for implementing the regional strategic plan for eliminating NTDs in the African region have been estimated at US$ 2.57 billion over a six-year period, which translates to US$ 428.33 million per year [101, 102]. This cost of implementing national-level “NTD Master Plans” for controlling NTDs is by far much lower than our estimated NTD-related productivity losses of Int$ 5.1 billion.

Third, effective medicines for treating all NTDs are available and a sufficient amount of donated drugs has been pledged by their manufacturers to meet the needs in endemic countries [102, 103].

Fourth, even though NTDs are not a major cause of death, every year they lead to a substantive loss of disability-adjusted life years (DALYs) in Africa. For example, the WHO estimated that in 2015, the African continent lost 10.3 million DALYs due to neglected parasitic diseases and intestinal nematodes [104].

Fifth, global and continental plans and programmes for combating NTDs exist. They provide detailed guidance on the cost-effective NTD interventions that individual countries should invest in. These plans include: the WHO global strategy 2015–2020 on water, sanitation and hygiene for accelerating and sustaining progress on neglected tropical diseases [105]; regional strategy on NTDs [106]; regional strategic plan for NTDs [106]; roadmap for accelerating work to overcome the global impact of neglected tropical diseases [107]; and the global plan to combat neglected tropical diseases 2008–2015 [108]. In 2015, the Expanded Special Project for Elimination of Neglected Tropical Diseases was established in Africa [109].

Sixth, continental and global political commitment exists for ending the morbidity and mortality from NTDs. In January 2014, the Twenty-Fourth Ordinary Session of the African Union Executive Council adopted the Continental Framework on the Control and Elimination of NTDs in Africa by 2020 and committed to using it for developing and revising national NTD plans [110]. In 2013, the World Health Assembly, through resolution WHA66.12, adopted a comprehensive global strategy for combatting NTDs [111]. In the same year, the Sixty-Third Regional Committee for Africa through resolution AFR/RC63/R6 adopted both the regional strategy and the strategic plan on NTDs [112]. The African Union decisions and WHO Regional Committee for Africa resolutions urge African countries and their partners to commit more resources and use them efficiently to implement the Continental Framework on the Control and Elimination of NTDs through national NTD plans.

### Limitations

There are seven broad limitations of the current study. First, some costs were omitted. For example, direct costs of NTD prevention programmes, and diagnosis and treatment services were not taken into account because the current study focused on years of life lost due to premature mortality. The study also excluded the indirect costs of productive labour time lost due to morbidity, including cost of time spent seeking treatment, reduced level of performance of activities/functions of daily living, and time expended by caregivers (family and friends) and those accompanying the sick to sources of care, e.g. health facilities, private pharmaceutical shops, traditional healers. The intangible/psychological costs related to stigmatisation, discrimination, pain, anxiety and bereavement were also omitted.

Second, to date there is no consensus in the published literature on the discount rate that should be applied in health sector studies. In this study, we used discount rate of 3%, which has been applied frequently in past health-related studies [81, 113–115].

Third, there is no agreement in literature about whether mortality occurring at different age groups should be weighted differently. In our study, we assumed all life to be intrinsically valuable and thus a year lost in an age group was considered to be of equal value [116]. This is why the current study used per-capita GDP to value YLLs at any age group.

Fourth, various authors have underscored a few weaknesses inherent in the use of per-capita GDP as a measure of societal economic and wellbeing: (a) per-capita GDP is an average value, which is distorted by high-income earners and corporate supernormal profits, and does not reflect distribution of income, consumption and wealth [117]. Therefore, if a country’s GDP distribution is skewed, a small wealthy class can increase per-capita GDP substantially while the majority of the population does not experience any economic and social progress [117]. (b) Per-capita GDP does not factor in the negative externalities of goods and services production and delivery processes, e.g. depletion of natural resources, air pollution from carbon emissions of airplanes and vehicles, global warming, and contamination of water with industrial waste [117]. (c) The value of household and other unpaid work is not measured in the system of national accounts that produces GDP. A substantial burden of unpaid domestic work (preparing food, cleaning and maintaining the home) and unpaid care work (care a person provides to their own family and household members) in Africa is borne by women [118–121]. We concur with Hirway [121] that the exclusion of unpaid domestic and caring work from national accounts and from the conventional economy is not justifiable, as both contribute to the conventional economy.

Fifth, a number of weaknesses characterise the lost output approach or HCA: (a) It assumes that the objective of health care is getting sick people back to productive employment. However, there are other objectives, such as preventing morbidity and death so that people can enjoy life (flourish), enjoy leisure and perform non-economic societal functions, etc. (b) In its pure form, the HCA would value the lives of pensioners (elderly), full-time homemakers and non-working children at zero. In this study, we value all lives using per-capita GDP prevailing in each country. (c) The HCA does not capture intangible psychological costs of NTDs, e.G. *stigma*, pain, bereavement, anxiety and suffering [122, 123].

Sixth, the values of life loss estimates reported in this paper are not a guide to setting priorities in the research, prevention and treatment of NTDs [124, 125]. The estimated value of human lives lost due to NTDs are only meant for use in raising public awareness and advocacy with ministries of finance in African countries on the magnitudes of potential economic losses arising from mortality associated with NTDs. Therefore, we are cognisant of the fact that setting priorities in NTD research, prevention and treatment must be guided by economic evaluation evidence on costs and consequences of competing research, prevention and treatment strategies [81, 82].

Seventh, it is common knowledge that vital registration systems in many countries in Africa are either non-existent or very weak. That is why the deaths and burden of disease estimates reported by international organisations are often projections based on second-best approaches. Thus, it is usually not possible to verify the coverage and quality of secondary mortality data among the analysed countries.

## Conclusions

Even though NTDs are not a major cause of death, they impact negatively on the productivity of those affected throughout their life-course. Thus, the case for investing in NTD control should also be influenced by the value of NTD morbidity, availability of effective donated medicines, human rights arguments and need to achieve the NTD-related target 3.3 of the UN SDG 3 (on health) by 2030.

In order for the African continent to have a fair chance of ending the epidemic of NTDs by 2030, as envisioned in SDG 3, the national governments, African Union, Regional Economic Communities and all partners need to continue fighting the war against NTDs until they are all controlled, eliminated, eradicated and eventually extinct from the continent. As long as corruption and lack of accountability remain endemic due to weak leadership [126–129] and governance [130–132], the war against NTDs (and other causes of ill-health) is unlikely to be won. Thus, African governments and development partners need to continue their efforts to fully implement the Paris Declaration on Aid Effectiveness and the Accra Agenda for Action [133] to ensure strategic policy frameworks exist and are combined with effective integration, coordination, oversight (to assure efficiency), coalition building, the provision of appropriate regulations and incentives, attention to system-design and accountability [134–136].

## Additional files

Additional file 1: Multilingual abstracts in the five official working languages of the United Nations. (PDF 392 kb)

Additional file 2: Number of NTD deaths in Africa in 2015. (DOCX 13 kb)

Additional file 3: WHO Global Health Estimates of Frontier Years of Life Lost (not age weighted or discounted). (DOCX 12 kb)

Additional file 4: Non-health GDP per capita. (DOCX 13 kb)

Additional file 5: Illustration of estimation of value of human life lost in a country. (DOCX 310 kb)

## Abbreviations

*GDPPC*_*Int*$_: Per capita GDP in international dollars
*NHGDPPC*_*Int*$_: Per capita non-health GDP in PPP
*VHLLost*_0 − 4_: Value of human lives lost among those aged 0–4 years
*VHLLost*_15 − 29_: Value of human lives lost among those aged 15–29 years
*VHLLost*_30 − 49_: Value of human lives lost among those aged 30–49 years
*VHLLost*_50 − 59_: Value of human lives lost among those aged 50–59 years
*VHLLost*_5 − 14_: Value of human lives lost among those aged 5–14 years
*VHLLost*_60 − 69_: Value of human lives lost among those aged 60–69 years
*VHLLost*_≥70_: Value of human lives lost among those aged 70 years and above
DALY: Disability-adjusted life year
DRC: Democratic Republic of the Congo
GDP: Gross domestic product
ILO: International Labour Organization
IMF: International Monetary Fund
Int$: International dollars
NTD: neglected tropical disease
NTDD: NTD death
PCTHE: Per-capita total health expenditure
PPP: Purchasing power parity
r: Rate of discount of future losses
UN: United Nations
*VHLLost*: Value of human lives lost
WHO: World Health Organization
YLL: Year of life lost
