# Supplementary material for: The monetary value of human lives lost due to neglected tropical diseases in Africa
Source: Infect Dis Poverty. 2017 Dec 18;6:165. doi: 10.1186/s40249-017-0379-y (PMC5733961; doi:10.1186/s40249-017-0379-y)
Supplement: Supplementary file 1 — Multilingual abstracts in the five official working languages of the United Nations. (PDF 392 kb) [file 40249_2017_379_MOESM1_ESM.pdf]

Translation of the abstract into the five official working languages of the United Nations

القيمة النقدية للأرواح البشرية التي فقدت بسبب أمراض المناطق المدارية المهملة في أفريقيا

خوسيس موثوري كيريجيا وجيتونجا ن. مبوروجو

ملخص

الخلفية: الأمراض المدارية المهملة سبب مهم للوفاة والعجز في أفريقيا. وتقدر هذه الدراسة القيمة النقدية للأرواح البشرية التي فقدت بسبب الأمراض القلبية الوخيمة في القارة في عام 2015.

الأساليب: استخدم الناتج المفقود أو نهج رأس المال البشري لتقييم سنوات الحياة المفقودة بسبب الوفيات المبكرة الناجمة عن الأمراض غير السارية بين 10 من ذوي الدخل المرتفع / الأعلى من الشريحة المتوسطة (المجموعة 1) و17 من الشريحة المتوسطة 2 (المجموعة 2) (المجموعة الثالثة) في أفريقيا. تم خصم الخسائر المستقبلية إلى قيمها الحالية بمعدل خصم بنسبة 3٪. تم إعادة تحليل النموذج باستخدام نسب خصم 5٪ و10٪ لتقييم التأثير على القيمة الإجمالية التقديرية للأرواح البشرية المفقودة.

النتائج: بلغت القيمة التقديرية لـ67860 من الأرواح البشرية المفقودة في عام 2015 بسبب الأمراض المدارية المهملة 5112472607 دولارا أمريكيا. ومن بين ذلك، تحملت المجموعة 1 14.6% وتحملت المجموعة الثانية 57.7% ودول المجموعة الثالثة 27.7%. وبلغت القيمة المتوسطة للحياة البشرية المفقودة في الوفيات الناجمة عن الأمراض القلبية الوعائية 231278 دولارا أمريكيا و109771 دولارا أمريكيا و37489 دولارا أمريكيا لدول المجموعة الأولى والمجموعة الثانية والمجموعة الثالثة على التوالي. وكانت القيمة التقديرية للأرواح البشرية المفقودة في عام 2015 بسبب الأمراض غير المعدية تعادل 0.1% من الناتج المحلي الإجمالي التراكمي لـ53 بلدا أفريقيا مداريا.

الخلاصة: على الرغم من أن الأمراض المدارية المهملة ليست سببا رئيسيا للوفاة، إلا أنها تؤثر سلبا على إنتاجية المتضررين طوال حياتهم. وبالتالي، ينبغي أن تتأثر حالة الاستثمار في مكافحة الأمراض القلبية الوخيمة أيضا بقيمة انتشار الأمراض القلبية الوخيمة، وتوافر الأدوية المتبرع بها الفعالة، والحجج المتعلقة بحقوق الإنسان، والحاجة إلى تحقيق الهدف 3,3 من أهداف التنمية المستدامة للأمم المتحدة الهدف 3 (بشأن الصحة) بحلول عام 2030.

Translated from English version into Arabic by Mahmoud Sami, through

TRANSLATORS WITHOUT BORDERS

非洲被忽视热带病所致人类寿命损失的货币价值

Joses Muthuri Kirigia and Gitonga N. Mburugu

摘要

引言：在非洲，被忽视热带病（NTDs）是致死和致残的一个重要原因。本研究估算了 2015 年非洲大陆 NTDs 所致人类寿命损失的货币价值。

方法：本研究采用产出损失或人力资本法来估算非洲国家因 NTDs 过早死亡而丧失的寿命年限。研究对象分为三组，10 个高中收入国家（第 1 组）、17 个中等收入国家（第 2 组）和 27 个低收入国家（第 3 组）。未来损失以 3%的贴现率贴至现值。该模型使用 5%和 10%的贴现率进行重新分析，以评估对估算的人类寿命损失总值的影响。

结果：2015 年 NTDs 所致的 67 860 人类寿命损失的估算总额为 Int$ 5 112 472 607。其中，第 1 组占 14.6%，第 2 组占 57.7%，第 3 组占 27.7%。上述三组国家 NTD 死亡病例的人均寿命损失平均值分别为：第 1 组 Int$ 231 278，第 2 组 Int$ 109 771，第 3 组 Int$ 37 489。据估算，2015 年非洲 NTDs 所致人类寿命损失的货币值相当于非洲大陆 53 个国家累计国内生产总值的 0.1%。

结论：尽管 NTDs 不是致死主因，但将严重影响患者终身的生产力。因此，投资 NTDs 控制措施也应考虑到 NTD 发病率、有效捐赠药物的可及性、人权论点以及 2030 年实现联合国可持续发展目标 3 （健康相关）的影响。

Translated from English version into Chinese by Jin Chen, edited by Pin Yang

CHINESE CENTER FOR DISEASE CONTROL AND PREVENTION
NATIONAL INSTITUTE OF PARASITIC DISEASES

Valeur monétaire des vies humaines perdues à cause des maladies tropicales négligées en Afrique

Joses Muthuri Kirigia et Gitonga N. Mburugu

Résumé

Contexte : Les maladies tropicales négligées (MTN) sont une cause importante de mortalité et de handicap en Afrique. Notre étude estime la valeur monétaire des vies humaines perdues à cause d'elles sur le continent en 2015.

Méthodes : Nous avons utilisé une approche par la perte de productivité ou de capital humain pour évaluer les années de vie perdues à cause des décès prématurés causés par les MTN dans 10 pays d'Afrique à revenus élevés (groupe 1) 17 à revenus moyens (groupe 2) et 27 à bas revenus (groupe 3). Les pertes futures ont été actualisées à leur valeur présente à un taux de 3 %. Le modèle a été réanalysé avec des taux d'actualisation de 5 % et 10 % afin d'évaluer l'impact sur la valeur totale estimée des vies humaines perdues.

Résultats : La valeur des 67 860 vies humaines perdues en 2015 à cause des MTN a été estimée à 5 112 472 607 Int$, dont 14,6 % dans les pays du groupe 1, 57,7 % dans le groupe 2 et 27,7 % dans le groupe 3. La valeur moyenne de la vie humaine perdue pour chaque décès causé par une MTN était de 231 278 Int$ dans le groupe 1, 109 771 dans le groupe 2 et 37 489 dans le groupe 3. La valeur estimée des vies humaines perdues à cause des MTN en 2015 équivalait à 0,1 % du PNB cumulé des 53 pays d'Afrique continentale.

Conclusion : Bien que les maladies tropicales négligées ne soient pas une cause majeure de mortalité, elles ont un impact négatif sur la productivité des personnes affectées pendant toute leur vie. L'intérêt d'un investissement dans la lutte contre ces maladies doit donc aussi être évalué en fonction de la valeur de la morbidité liée aux MTN, de la disponibilité de dons de médicaments efficaces, de considérations sur les droits humains et de la nécessité d'atteindre, d'ici 2030, le but visé au point 3.3. du troisième Objectif de développement durable des Nations Unies (« Permettre à tous de vivre en bonne santé ») relatif aux MTN.

Translated from English version into French by Tatyana Johnson, through

TRANSLATORS
WITHOUT BORDERS

Монетарная стоимость потерянных человеческих жизней в результате «забытых» тропических болезней в Африке

Жозе Мутури Киригиа (Joses Muthuri Kirigia) и Гитонга Н. Мбуругу (Gitonga N. Mburugu)

Реферат

Предыстория: Забытые тропические болезни (ЗТБ) являются распространенной причиной смерти и потери дееспособности в Африке. В данном исследование дается оценка монетарной стоимости человеческих жизней, потерянных вследствие забытых тропических болезней на континенте в 2015 году.

Методы: Оценка потерянных лет жизни в результате преждевременной смерти из-за забытых тропических болезней проводилась среди следующих групп африканских стран: 10 стран с высоким/средним уровнем доходов (Группа 1), 17 стран со средним уровнем доходов (Группа 2) и 27 стран с низким уровнем доходов (Группа 3). При этом использовалась концепция потерь производительности, или человеческого капитала. Будущие потери были дисконтированы до их существующих показателей при ставке дисконтирования 3%. Для оценки воздействия на ожидаемую общую стоимость потерянных человеческих жизней модель была повторно проанализирована с использованием ставок дисконтирования 5% и 10%.

Результаты: Ожидаемая стоимость 67 860 человеческих жизней, утраченных в 2015 году вследствие забытых тропических болезней, составила в международных долларах 5 112 472 607. Из этой суммы страны Группы 1 несли 14,6% расходов, Группы 2 – 57,7% и Группы 3 – 27,7%. Средняя стоимость потерянных человеческих жизней в результате забытых тропических болезней составила в международных долларах 231 278, 109 771 и 37 489 для стран Группы 1, Группы 2 и Группы 3 соответственно. Ожидаемая стоимость человеческих жизней, потерянных в 2015 году в результате забытых тропических болезней, равнялась 0,1% совокупного валового внутреннего продукта 53 стран континентальной Африки.

Выводы: Хотя забытые тропические болезни не составляют основную причину смерти, они негативно влияют на производительность лиц, затронутых этими заболеваниями, на протяжении всей их жизни. Таким образом, необходимость инвестирования в борьбу с забытыми тропическими болезнями будет также определяться такими факторами, как: показатель заболеваемости ЗТБ, доступность предоставляемых на безвозмездной основе эффективных медикаментов, правозащитные аргументы и необходимость достижения связанного с ЗТБ целевого показателя 3.3 Цель 3 (здравоохранение) программы ООН Цели в области устойчивого развития до 2030 г.

Translated from English version into Russian by Tatyana Johnson, through

El valor monetario de las vidas humanas se pierde debido a las enfermedades tropicales desatendidas en África

Joses Muthuri Kirigia i Gitonga N. Mburugu

Abstracto

Antecedentes: las enfermedades tropicales desatendidas (ETD) son una causa importante de muerte y discapacidad en África. Este estudio estima el valor monetario de vidas humanas perdidas debido a las ETD en el continente en 2015.

Métodos: Se utilizó el enfoque de producción perdida o de capital humano para evaluar los años de vida perdidos debido a muertes prematuras debidas a ETD entre 10 ingresos de ingresos alto / medio alto (Grupo 1), 17 ingresos medios (Grupo 2) y 27 ingresos bajos países de ingresos (grupo 3) en África. Las pérdidas futuras se descontaron a sus valores actuales a una tasa de descuento del 3%. El modelo se volvió a analizar utilizando tasas de descuento del 5% y 10% para evaluar el impacto en el valor total estimado de vidas humanas perdidas.
Resultados: El valor estimado de 67 860 vidas humanas perdidas en 2015 por ETD fue de $ 5 112 472 607. Fuera de eso, el Grupo 1 sufrió el 14.6%, el Grupo 2 el 57.7% y el Grupo 3 el 27.7%.El valor medio de la vida humana perdida por muerte ETD fue de Int $ 231 278, Int $ 109 771 e Int $ 37 489 para los países del Grupo 1, Grupo 2 y Grupo 3, respectivamente. El valor estimado de las vidas humanas perdidas en 2015 debido a las ETD equivalía al 0,1% del producto interno bruto acumulado de los 53 países de África continental
Conclusión: aunque las ETD no son una causa importante de muerte, tienen un impacto negativo en la productividad de los afectados a lo largo de su curso de vida. Por lo tanto, el caso de invertir en el control de las ETD también debe verse influenciado por el valor de la morbilidad de lasETD, la disponibilidad de medicamentos donados efectivos, los argumentos de derechos humanos y la necesidad de alcanzar el objetivo 3,3 relacionado con la ETD del Objetivo 3 de Desarrollo Sostenible de las Naciones Unidas(salud) para 2030.

Translated from English version into Spanish by sahar05, through

TRANSLATORS
WITHOUT BORDERS
