# Supplementary material for: The monetary value of human lives lost due to neglected tropical diseases in Africa
Source: Infect Dis Poverty. 2017 Dec 18;6:165. doi: 10.1186/s40249-017-0379-y (PMC5733961; doi:10.1186/s40249-017-0379-y)
Supplement: Supplementary file 2 — Number of NTD deaths in Africa in 2015. (DOCX 13 kb) [file 40249_2017_379_MOESM2_ESM.docx]

| Additional File 2: Number of NTD deaths in Africa in 2015 | |
| --- | --- |
| Countries | NTD Deaths 2015 |
| Algeria | 252 |
| Angola | 1,854 |
| Benin | 599 |
| Botswana | 22 |
| Burkina Faso | 1,088 |
| Burundi | 751 |
| Cameroon | 1,505 |
| Cape Verde | 4 |
| Central African Republic | 742 |
| Chad | 1,659 |
| Comoros | 20 |
| Congo | 175 |
| Cote d'Ivoire | 2,758 |
| Democratic Republic of Congo | 7,298 |
| Djibouti | 34 |
| Egypt | 630 |
| Equatorial Guinea | 47 |
| Eritrea | 279 |
| Ethiopia | 7,315 |
| Gabon | 107 |
| Gambia | 68 |
| Ghana | 1,150 |
| Guinea | 1,271 |
| Guinea-Bissau | 95 |
| Kenya | 1,469 |
| Lesotho | 29 |
| Liberia | 192 |
| Libya | 35 |
| Madagascar | 1,017 |
| Malawi | 554 |
| Mali | 1,009 |
| Mauritania | 154 |
| Mauritius | 7 |
| Morocco | 175 |
| Mozambique | 1,543 |
| Namibia | 18 |
| Niger | 1,428 |
| Nigeria | 13,944 |
| Rwanda | 408 |
| Sao Tome and Principe | 3 |
| Senegal | 474 |
| Seychelles | 1 |
| Sierra Leone | 665 |
| Somalia | 851 |
| South Africa | 882 |
| South Sudan | 3,013 |
| Sudan | 4,184 |
| Swaziland | 23 |
| Togo | 443 |
| Tunisia | 34 |
| Uganda | 2,344 |
| Tanzania | 2,278 |
| Zambia | 618 |
| Zimbabwe | 346 |
| TOTAL | 67,860 |
