# Supplementary material for: The monetary value of human lives lost due to neglected tropical diseases in Africa
Source: Infect Dis Poverty. 2017 Dec 18;6:165. doi: 10.1186/s40249-017-0379-y (PMC5733961; doi:10.1186/s40249-017-0379-y)
Supplement: Supplementary file 3 — WHO Global Health Estimates of Frontier Years of Life Lost (not age weighted or discounted). (DOCX 12 kb) [file 40249_2017_379_MOESM3_ESM.docx]

Additional File 3: WHO Global Health Estimates of Frontier Years of Life Lost (not age weighted or discounted)

| Age range | Years of Life Lost | Age Group (years) | Years of Life Lost |
| --- | --- | --- | --- |
| Neonatal | 91.93 | 0-4 | 91 |
| Postneonatal | 91.55 |  |  |
| 1-4 | 89.41 |  |  |
| 5-9 | 84.52 | 5-14 | 82 |
| 10-14 | 79.53 |  |  |
| 15-19 | 74.54 | 15-29 | 70 |
| 20-24 | 69.57 |  |  |
| 25-29 | 64.60 |  |  |
| 30-34 | 59.63 | 30-49 | 52 |
| 35-39 | 54.67 |  |  |
| 40-44 | 49.73 |  |  |
| 45-49 | 44.81 |  |  |
| 50-54 | 15.65 | 50-59 | 37 |
| 55-59 | 12.82 |  |  |
| 60-64 | 30.25 | 60-69 | 28 |
| 65-69 | 25.49 |  |  |
| 70-74 | 20.77 | 70+ | 14 |
| 75-79 | 16.43 |  |  |
| 80-84 | 12.51 |  |  |
| 85+ | 7.60 |  |  |

Source: WHO []
