# Supplementary material for: The monetary value of human lives lost due to neglected tropical diseases in Africa
Source: Infect Dis Poverty. 2017 Dec 18;6:165. doi: 10.1186/s40249-017-0379-y (PMC5733961; doi:10.1186/s40249-017-0379-y)
Supplement: Supplementary file 4 — Non-health GDP per capita. (DOCX 13 kb) [file 40249_2017_379_MOESM4_ESM.docx]

| Additional File 4: Non-health GDP per capita | |
| --- | --- |
| Country | Non-Health PCPGDP (Int$) in 2015 |
| Algeria | 13,852 |
| Angola | 6,695 |
| Benin | 2,092 |
| Botswana | 16,120 |
| Burkina Faso | 1,731 |
| Burundi | 764 |
| Cameroon | 3,139 |
| Cape Verde | 6,340 |
| Central African Republic | 626 |
| Chad | 2,482 |
| Comoros | 1,416 |
| Congo, Democratic Republic of | 739 |
| Congo, Republic of | 6,420 |
| Côte d'Ivoire | 3,370 |
| Equatorial Guinea | 37,598 |
| Eritrea | 1,258 |
| Ethiopia | 1,840 |
| Gabon | 18,774 |
| Gambia, The | 1,519 |
| Ghana | 4,286 |
| Guinea | 1,201 |
| Guinea-Bissau | 1,497 |
| Kenya | 3,167 |
| Lesotho | 2,842 |
| Liberia | 747 |
| Madagascar | 1,480 |
| Malawi | 1,030 |
| Mali | 2,140 |
| Mauritania | 4,226 |
| Mauritius | 19,532 |
| Mozambique | 1,104 |
| Namibia | 10,735 |
| Niger | 1,056 |
| Nigeria | 5,695 |
| Rwanda | 1,771 |
| São Tomé and Príncipe | 3,107 |
| Senegal | 2,459 |
| Seychelles | 27,345 |
| Sierra Leone | 1,421 |
| South Africa | 11,980 |
| Swaziland | 9,203 |
| Tanzania | 2,945 |
| Togo | 1,456 |
| Uganda | 1,934 |
| Zambia | 3,687 |
| Zimbabwe | 1,848 |
| South Sudan | 1,530 |
| Djibouti | 2,865 |
| Egypt | 11,485 |
| Libya | 13,605 |
| Morocco | 7,874 |
| Somalia | Missing Data |
| Sudan | 4,157 |
| Tunisia | 10,895 |

Sources: WHO [ ] and IMF [ ]
