# Supplementary material for: The monetary value of human lives lost due to neglected tropical diseases in Africa
Source: Infect Dis Poverty. 2017 Dec 18;6:165. doi: 10.1186/s40249-017-0379-y (PMC5733961; doi:10.1186/s40249-017-0379-y)
Supplement: Supplementary file 5 — Illustration of estimation of value of human life lost in a country. (DOCX 310 kb) [file 40249_2017_379_MOESM5_ESM.docx]

**Additional File 5: Illustration of estimation of value of human life lost in a country**

It is demonstrated below, using actual data on Egypt, how to calculate the value of human life lost due to NTD deaths:

1. Total NTD deaths in 2015 = 630
2. Ratio of deaths among persons aged 0-4 years = 0.0440377242929898
3. Ratio of deaths among persons aged 5-14 years = 0.101634283536101
4. Ratio of deaths among persons aged 15-29 years = 0.1679551894939
5. Ratio of deaths among persons aged 30-49 years = 0.274857756724387
6. Ratio of deaths among persons aged 50-59 years = 0.128631602442755
7. Ratio of deaths among persons aged 60-69 years = 0.146513806413979
8. Ratio of deaths among persons aged 70+ years = 0.136369637095889
9. $NTDD_{0-4}$ = 630 x 0.0440377242929898 = 27.7580936442442
10. $NTDD_{5-14}$ = 630 x 0.101634283536101 = 64.0626645711993
11. $NTDD_{15-29}$ = 630 x 0.1679551894939 = 105.866412328455
12. $NTDD_{30-49}$ = 630 x 0.274857756724387 = 173.249809623268
13. $NTDD_{50-59}$ = 630 x 0.128631602442755 = 81.079758855377
14. $NTDD_{60-69}$ = 630 x 0.146513806413979 = 92.3513651967093
15. $NTDD_{\geq70}$ = 630 x 0.136369637095889 = 85.9572381977483
16. Mean age at death among persons aged 0-4 years ($AAD_{0-4}$), i.e. (0+4)/2 = 2 years
17. In order to reach the legal minimum age for employment of 15 years, one needs to add 12 years to the result in (p) = $Age_{0-4Min}$.
18. Mean age at death among persons aged 5-14 years ($AAD_{5-14}$), i.e. (5+14)/2 = 9.5 years
19. In order to reach the legal minimum age for employment of 15 years, one needs to add 4.5 years to the result in (r) =$Age_{5-14Min}$.
20. Per capita gross domestic product in 2016() = Int$ 12137.022
21. Per capita total expenditure on health () = Int$ 651.890651425544
22. 12137.022 - Int$ 651.890651425544 = Int$ 11485.1313485745
23. Discount rate ($r$) = 3%
24. Undiscounted years of life lost in the group aged 0-4 years ($YLL_{0-4}$) = LE – ($AAD_{0-4}+ Age_{0-4Min}$)= 91 – (2 + 12) = 77 years
25. Discounted years of life lost in the group aged 0-4 years ($DYLL_{0-4}$) = 29.91028964
26. Undiscounted years of life lost in the group aged 5-14 years ($YLL_{5-14}$) = LE – ($AAD_{5-14}+ Age_{5-14Min}$)= 82 – (9.5 + 4.5) = 68 years
27. Discounted years of life lost in the group aged 5-14 years ($DYLL_{5-14}$) = 28.86703771
28. Undiscounted years of life lost in the group aged 15–29 years ($YLL_{15-29}$) = 70 years
29. Discounted years of life lost in the group aged 15–29 years ($DYLL_{15-29}$) = 29.12342135 years
30. Undiscounted years of life lost in the group aged 30–49 years ($YLL_{30-49}$) = 52 years
31. Discounted years of life lost in the group aged 30–49 years ($DYLL_{30-49}$) = 26.16623999
32. Undiscounted years of life lost in the group aged 50–59 years ($YLL_{50-59}$) = 37 years
33. Discounted years of life lost in the group aged 50–59 years ($DYLL_{50-59}$) = 22.16723544 year
34. Undiscounted years of life lost in the group aged 60–69 years ($YLL_{60-69}$) = 28 years
35. Discounted years of life lost in the group aged 60-69 years ($DYLL_{60-69}$) = 18.76410823
36. Undiscounted years of life lost in the group aged 70+ years ($YLL_{70\pm}$) = 14 years
37. Discounted years of life lost in the group aged 70+ years ($DYLL_{70+}$) = 11.29607314 year
38. =  x x = 29.91028964 x 11485.1313485745 x 27.7580936442442 = Int$ 9 535 560
39. =  x x  = 28.86703771 x 11485.1313485745 x 64.0626645711993 = Int$ 21 239 446
40. =  x x = 29.12342135 x 11485.1313485745 x 105.866412328455 = Int$ 35 410 867
41. =  x x = 26.16623999 x 11485.1313485745 x 173.249809623268 = Int$ 52 065 501
42. =  x x = 22.16723544 x 11485.1313485745 x 81.079758855377 = Int$ 20 642 389
43. =  x x = 18.76410823 x 11485.1313485745 x 92.3513651967093 = Int$ 19 902 481
44. $VHLLost_{70}$ = $DYLL_{70}$ x $NHGDPPC_{ Int\$}$x $NTDD_{70}$= 11.29607314 x 11485.1313485745 x 85.9572381977483 = Int 11 151 824

Int$ 9535560 + Int$21239446 + Int$35410867 + Int$52065501 + Int$20642389 + Int$19902481 + Int$11151824= Int$ 169 948 068.
